# Impact of Voice Therapy on Pediatric Patients With Dysphonia and Vocal Nodules: A Systematic Review

**DOI:** 10.7759/cureus.24433

**Published:** 2022-04-24

**Authors:** Mohammad Al-Kadi, Mohamed A Alfawaz, Fahad Z Alotaibi

**Affiliations:** 1 Otolaryngology - Head & Neck Surgery, King Abdulaziz Medical City, Riyadh, SAU; 2 Otolaryngology - Head & Neck Surgery, Prince Sultan Military Medical City, Riyadh, SAU; 3 College of Medicine, Imam Mohammad Ibn Saud Islamic University (IMSIU), Riyadh, SAU

**Keywords:** voice therapies, voice therapy, pediatric, dysphonia, vocal nodules

## Abstract

One of the most prevalent pediatric ailments around the world is voice disorders. Around 5-million children suffer from voice disorders, and three out of five of them suffer from vocal nodule-induced persistent dysphonia. Nineteen out of 20 otolaryngologists recommend voice therapies for the treatment of pediatric vocal fold nodules. However, the benefits of these therapies still remain to be assessed systematically. The objective of this study is to systematically review the impact of voice therapy (direct and indirect) on pediatric patients with vocal nodules.

In this systematic review of randomized control trials (RCTs), four electronic databases, PubMed, CENTRAL (Cochrane), Science Direct, and Lancet, were explored for the literature survey. The impact of direct and indirect voice therapies on pediatric cases with vocal nodules was reviewed based on the results of the selected articles.

Based on stringent inclusion and exclusion criteria, six articles were selected. All these studies examined the effects of direct and indirect voice therapies on two types of voice disorders, that is, dysphonia and vocal nodules. Only one of the six studies reported significant alleviation of the patient condition post-intervention. However, none of the studies discussed the clinical significance of the interventions.

Three of the six included studies used both direct and indirect voice therapies and reported substantial differences in the data collected before and after the interventions. However, overall, the studies reported more significant improvements in patient conditions. More studies in this domain are still warranted, especially to help understand and define the meaning of the term “effectiveness” with respect to voice therapies.

## Introduction and background

Vocal nodules refer to the benign growths that form in the middle portion of membranous vocal folds. They often affect the voice quality, making it rough, breathy, scratchy, or hoarse. Pediatric cases with vocal nodules have been reported to exhibit vocal fatigue and often complain of throat pain. Speech-language pathologists (SLPs) offer therapy for vocal fold nodules. These therapies often comprise good vocal hygiene, treatment to counter changes in pitch and loudness, etc. Patients with vocal nodules are recommended to avoid smoke and other known allergens and to avoid straining their voices. Around 5-million children around the world are affected by voice disorders, of which at least 35-78% of children harbor vocal nodules or suffer from dysphonia, with a higher incidence in males. It is noteworthy that pediatric cases who suffer from vocal disorders often find it difficult to express themselves adequately. It often leads to underdeveloped communication skills and psychosocial inabilities that are often associated with poor self-esteem. Studies also show children with unresolved voice disorders can require additional ongoing treatment into adulthood, placing a substantial burden on the medical system [1].

Several recent files in this domain have started focusing on the voice therapies that are recommended by otolaryngologists for 95% of pediatric cases of vocal nodules. However, to the best of our knowledge, none of the studies have assessed the benefits of these therapies. In general, voice therapies are divided into two broad classes: direct voice therapy and indirect voice therapy. Direct voice therapies, as the name suggests, are directly focused on the “defective” region of the vocal system, including respiratory, muscular, skeletal, etc. These therapies directly modify the vocal behavior to induce healthy voice generation. Usually, direct therapies comprise vocal function exercises, resonance therapy, and semi-occlusion of the vocal tract. On the other hand, indirect voice therapies are more focused on altering the psychology and behavior of the patient and their surrounding environment. It mainly involves educating and counseling the patients regarding maintaining a healthy and hygienic vocal system and its advantages.

The objective of the current systematic review was to assess and compare the efficacies of the direct and indirect voice therapies among the pediatric cases of vocal nodules, so as to provide clinicians with a list of evidence-based voice therapy techniques.

## Review

Methods

Literature Search and Data Sources

The Preferred Reporting Items for Systematic Reviews and Meta-Analyses (PRISMA) guidelines were used to oversee the systematic literature review. The relevant studies were searched across four databases: PubMed, CENTRAL (Cochrane), ScienceDirect, and Lancet. The primary search queries used were “direct voice therapy”, “indirect voice therapy”, and “vocal nodules”. Furthermore, to avoid any discrepancy, the bibliography of the relevant articles was also screened. Table 1 describes the PICO (P - Population; I - Intervention; C - Comparator; O - Outcome(s)) method used in this study, and Table 2 describes the search strategies used for the different databases.

**Table 1:** PICO method used in this study PICO: P - Population; I - Intervention; C - Comparator; O - Outcome(s)

PICO	
Population	Children With Vocal Nodules
Intervention	Direct Voice Therapy
Comparator	Indirect Voice Therapy
Outcome	Pediatric Quality of Life Scale (PVRQOL) or GRBAS (Grade, Roughness, Breathiness, Asthenia, and Strain) Scale or Consensus Auditory Perceptual Evaluation of Voice (CAPE-V)

**Table 2:** Search strategies used for the different databases

Database	Search Engines	Result
PubMed	(Vocal) OR (Larynx)) OR (Laryngeal)) OR (Voice)) OR (Nodules))) AND (Therapy)) OR (Voice Assessment)) OR (Indirect Therapy)) AND (Direct Therapy)) AND (Indirect and Direct Therapy)) OR (Voice Outcome))	670
ScienceDirect	"Vocal Nodules" OR ("Larynx" OR "Laryngeal") AND "Voice Assessment" OR "Indirect Therapy" AND "Direct Therapy" AND "Indirect and Direct Therapy" OR "Voice Outcome"	96
CENTRAL (Cochrane)	"Vocal Nodules" OR "Larynx" OR "Laryngeal" AND "Voice Assessment" OR "Indirect Therapy" AND "Direct Therapy" AND "Indirect and Direct Therapy" OR "Voice Outcome"	178
Lancet	"Vocal" OR "Larynx" OR "Laryngeal" OR "Voice" OR "Nodules" AND "Therapy" OR "Voice Assessment" OR "Indirect Therapy" AND "Direct Therapy" AND "Indirect and Direct Therapy" OR "Voice Outcome"	172

Eligibility Criteria

Our study included a plethora of eligibility criteria across various domains, including the overall study design, methods used in the studies, and outcomes (Table 3).

**Table 3:** Eligibility criteria in our study

Inclusion Criteria	Exclusion Criteria
Publication	
Written in the English language	Dissertations, editorials, doctorates, and gray literature
Published between January 2001 and May 2021	Difficult to access articles
Peer-reviewed articles published in an indexed scientific journal	Incomplete trials
Patient cohort	
Studies comprising cohort aged <18 years	Studies comprising cohort aged >18 years
	Participants in the studies previously underwent voice therapy
	Animal or cadaver studies
Intervention	
Studies including direct and indirect voice therapies as interventions (irrespective of duration, intensity, or type of voice therapy)	All studies including therapies other than direct and indirect voice therapies
		Design	
		Randomized controlled trials (RCTs), cluster RCTs, quasi-RCTs, and randomized cross-over trials	Tool development studies
Interventions comprise ≥5 participants per group	Validity studies
		Outcome measures	
Articles that indicated the use of scale-related voice quality measures such as self-reported measures, observer-rated measures, and instrumental measures	-

Study Selection

The screening process began with eliminating the duplicate studies using the Zotero reference manager (Corporation for Digital Scholarship, Vienna, VA). Next, we used the Rayyan software (Rayyan Systems Inc., Cambridge, MA) for the initial screening of the remaining studies (reliability: 100%), and the remaining studies for review were included in the screening stages. We screened the titles and abstracts of the studies to filter out relevant studies based on the inclusion and exclusion criteria. The remaining studies were then subjected to a more stringent screening process via full-text review. The filtering of the studies depended on factors mentioned in Table 4.

**Table 4:** Factors affecting the filtering of studies

Title and abstract screening	Full-text screening
cohort with age > 18 years	no details regarding the voice therapy intervention used
animal studies	
vocal fold ailments other than nodules	
did not use voice therapy as the intervention	comprising other ailments and therapies
non-English	
editorials	
outside inclusion criteria period	

Quality Assessment

All the remaining studies were subjected to the Cochrane Risk of Bias (Cochrane Collaboration, London, United Kingdom) assessment using the Covidence® software (Veritas Health Innovation, Melbourne, Australia). Two reviewers checked for any discrepancies in the included studies while applying the critical appraisal tool.

Results

Search Result

**Figure 1:**
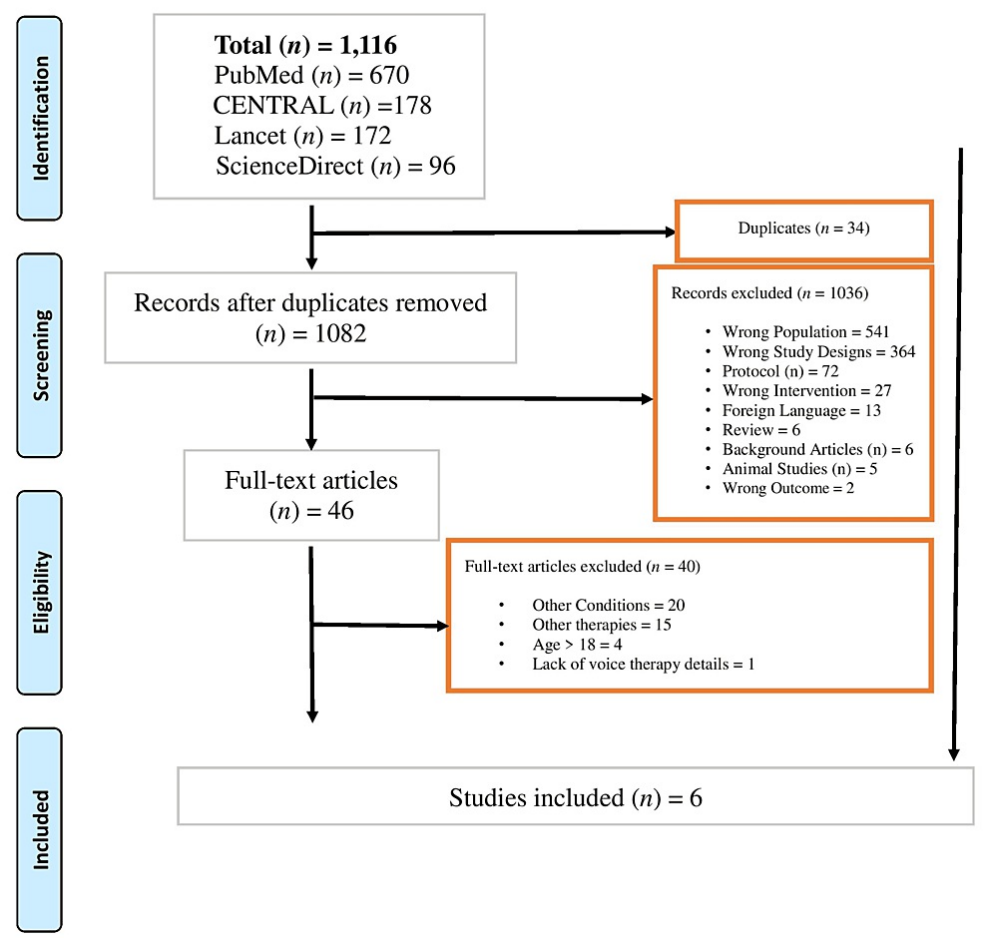
PRISMA flow diagram of the initial search strategy and screening process PRISMA: Preferred Reporting Items for Systematic Reviews and Meta-Analyses

The initial search strategy brought forth 1116 articles. Removal of duplicates reduced the number to 1036 articles. Among them, a total of six studies involved the use of direct and indirect voice therapies. Figure 1 depicts a PRISMA flow chart representing the search strategy and screening process.

Effect of Direct and Indirect Voice Therapy

Among the six studies, four employed the Grade, Roughness, Breathiness, Asthenia, and Strain (GRBAS) scale, one employed the Pediatric Quality of Life Scale, and one utilized the Consensus Auditory Perceptual Evaluation of Voice scale to assess the voice-related quality of life among pediatric cases with vocal nodules.

Of the six studies, two studies involved direct voice therapy as the intervention in pediatric cases with vocal nodules. These studies reported a significant difference in the voice quality of the patients after the therapy. One study used only indirect voice therapy and reported significant improvement among the participants post-intervention. Three studies used both direct and indirect voice therapies. One of these three studies reported significant improvement in patient outcomes following direct voice therapy. Another of these three studies did not find any significant differences between responses resulting from direct and indirect therapies.

Study Characteristics

We extracted a plethora of information from the included studies such as year and place of study, study type, sample size, inclusion and exclusion criteria, duration, key findings, etc. Overall, the studies recruited individuals of ages six to 16 years. The total sample size varied from 16 to 114 with an average of 65 cases. Of the six studies, five had a higher proportion of males, while one study had an equal number of males and females.

Quality Assessment

**Figure 2:**
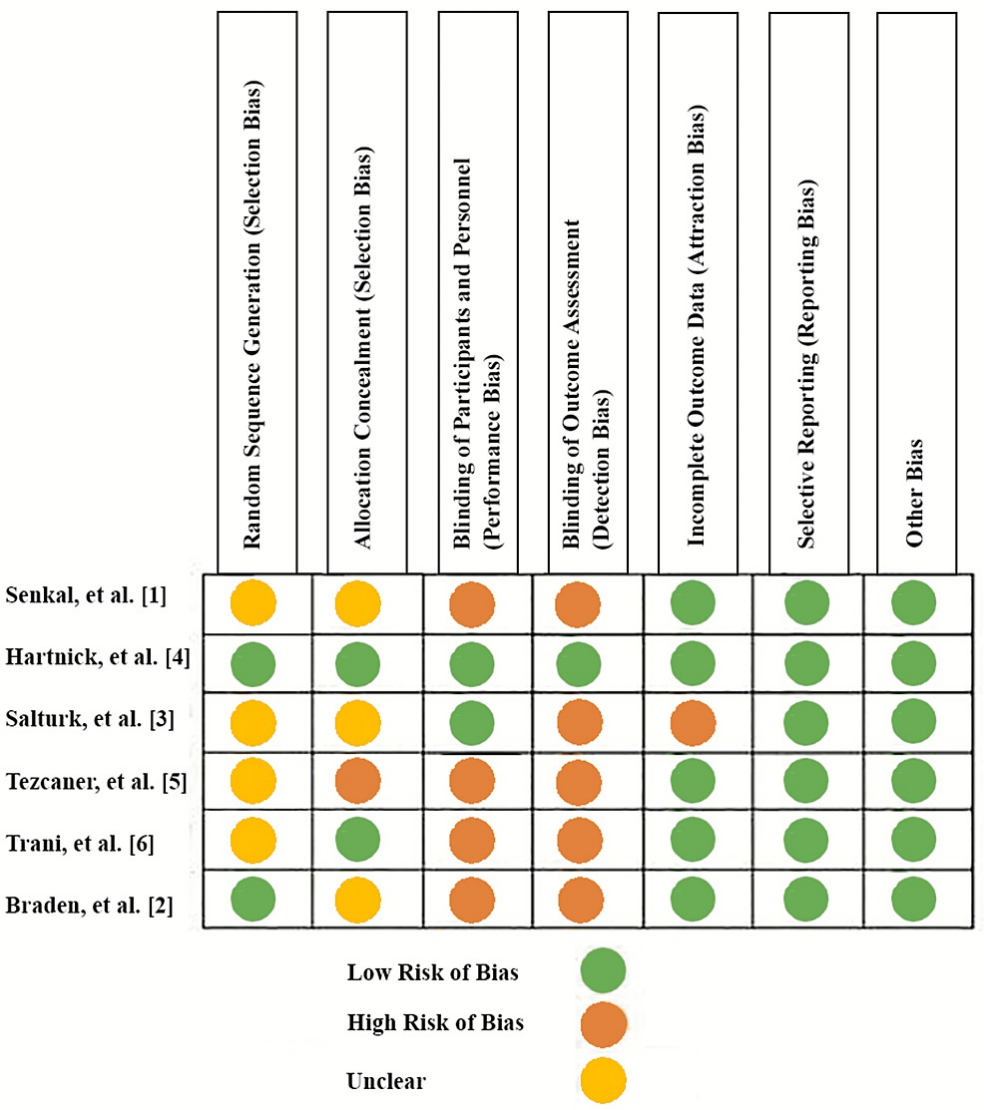
Risk of bias assessment Other biases include Selection bias, Information bias, and Confounding bias. [1-6]

As shown in Figure 2, most of the included studies showed a moderate risk of bias. Three studies did not describe the method of sequence generation. Three studies did not describe the methods for allocation concealment. Blinding of participants and personnel was described in two studies. Blinding of outcome was described in only one study. Only one study has incomplete outcome data, and all the studies have a low risk of reporting bias and other biases.

Discussion

Overall, our results indicated that studies on the application of voice therapies in pediatric cases with vocal nodules, to date, are riddled with methodology issues. For instance, some studies use objective while others use subjective instruments to measure the outcome of the voice therapies. This poses a problem with respect to sound statistical analysis. Furthermore, the studies often recruit a small number of participants, therapists, and/or instruments, which limits the potential of their data. The above-mentioned restrictions limit one’s ability to draw any definite conclusions; however, one may observe the relevant trends in this domain. For instance, in general, direct voice therapies have been observed to be more effective than indirect ones. It is noteworthy that it is still too early to determine which therapy is more effective in actual terms owing to a plethora of variables such as patient characteristics, type of therapy used, adequacy of diagnoses, and the type of instrument used to measure the voice-related quality of life.

Both Therapies

Three of the six included studies used both direct and indirect voice therapies for their cohort. In all these three studies, the patient outcome was reported to improve substantially, irrespective of the type of therapy used. One of these three studies was conducted in Turkey and reported significant changes in the Maximum Phonation Time (MPT) and GRBAS score of the cohort post-intervention, irrespective of the voice therapy used. They found symptomatic voice therapy to be the most effective [2]. Another study was conducted more recently on children suffering from dysphonia. They assessed the acoustic, aerodynamic, and perceptual measures before and after therapy and reported a significant difference in the phonation threshold pressure and the perceptual rating pre and post-intervention [3]. Another one of these studies was a multicentric RCT that recruited cases with both vocal nodules and dysphonia. The investigators here reported a significant improvement in the voice-related quality of life of the patients post-intervention; however, they did not observe a significant difference in the outcomes of the patients with respect to the type of therapy [1].

Direct Voice Therapy

Of the six studies, two studies used only direct voice therapy. One of these studies used resonance therapy on pediatric cases with vocal nodules. They used the GRBAS scale, pediatric voice handicap index (Turkish version), and acoustic voice analysis for outcome assessment and reported a success rate of 86%. Overall, they observed a marked difference in their data pre and post-intervention [4]. The other study employed Barragan’s method associated with S. Magnani’s vocal counseling for children with dysphonia. They conducted a phoniatric and psychological evaluation for all the patients and reported an improvement in 69% of the patients, with 44% of patients completely healed. They observed that the direct therapy did not affect the electro-acoustical parameters but there was a significant improvement in the MPT of the patients [5].

Indirect Voice Therapy

Of the six selected studies, one study employed indirect voice therapy for pediatric cases with vocal nodules. In this study, the patients were subjected to subjective assessment and acoustic assessment. They also employed the GRBAS scale and multi-dimensional voice program (MDVP) for outcome assessment. They observed that a tailored therapy could lead to substantial improvement in the outcome of the patients. They also reported that the GRBAS scale and acoustic analysis could be successfully used to assess patient condition during follow-up [6].

Owing to the availability of fewer studies and the small sample size and heterogeneity of the studies, it is not possible to determine the effectiveness of the voice therapy programs definitively [7-10]. More studies are still warranted in this domain to further elucidate the specific components of these therapies that impact the patient outcome most significantly. Such information could be useful while tailoring the therapies according to the patients. It is recommended that future studies in this area should comprise a larger cohort and application of an intervention on the control group (to eliminate the placebo effect).

Study limitations

The present study had a few limitations. Only the articles written in the English language were included and even the gray literature on this topic was ignored. The number of studies included in this review was very small, which eliminates any odds of conducting a pooled meta-analysis to further validate the efficiencies of voice therapies. The included studies were heterogeneous with respect to the research design and details surrounding the voice therapy employed. Finally, the included studies lacked any standardization of the outcome measures, which makes it difficult to draw any useful comparisons among the studies.

## Conclusions

Three of the six included studies used both direct and indirect voice therapies and reported substantial differences in the data collected before and after the interventions. However, overall, the studies reported more significant improvements in patient conditions. More studies in this domain are still warranted, especially to help understand and define the meaning of the term “effectiveness” with respect to voice therapies.
